# Giant Cell Arteritis: State of the Art in Diagnosis, Monitoring, and Treatment

**DOI:** 10.5041/RMMJ.10496

**Published:** 2023-04-30

**Authors:** Abid Awisat, Shiri Keret, Amal Silawy, Lisa Kaly, Itzhak Rosner, Michael Rozenbaum, Nina Boulman, Aniela Shouval, Doron Rimar, Gleb Slobodin

**Affiliations:** 1 Rheumatology Unit, Bnai Zion Medical Center, Haifa, Israel; 2 Rheumatology Clinic, Maccabi Health Services, Haifa, Israel

**Keywords:** Diagnosis, giant cell arteritis, monitoring, treatment

## Abstract

Giant cell arteritis (GCA) is the most prevalent subtype of vasculitis in adults. In recent years, there has been substantial improvement in the diagnosis and treatment of GCA, mainly attributed to the introduction of highly sensitive diagnostic tools, incorporation of modern imaging modalities for diagnosis and monitoring of large-vessel vasculitis, and introduction of highly effective novel biological therapies that have revolutionized the field of GCA. This article reviews state-of-the-art approaches for the diagnosis, monitoring, and treatment options of GCA.

## INTRODUCTION

Giant cell arteritis (GCA) is a granulomatous subtype of vasculitis involving large and medium-size arteries, both cranial and extra-cranial, e.g. aorta, temporal, subclavian, and axillary arteries. Giant cell arteritis is the most common form of vasculitis prevalent in adults after the age of 50, with the highest incidence observed in people from Northern Europe and of Scandinavian descent (14.6 to 43.6 per 100,000 aged >50 years)^1^ compared to lower incidence rates in Asia, Africa, and other parts of the world.^2^,^3^

The recently published 2022 European League Against Rheumatism (EULAR)/American College of Rheumatology (ACR) classification criteria for GCA have a sensitivity of 87.0% (95% CI 82.0%–91.0%) and a specificity of 94.8% (95% CI 91.0%–97.4%) as compared to a sensitivity of only 37.1% for the 1990 ACR criteria in the diagnosis of GCA with large vessel involvement.^4^,^5^ However, to date, no diagnostic criteria for GCA have been officially established.

This impressive improvement in the diagnosis of GCA throughout the last decades can be attributed to the introduction of highly sensitive diagnostic tools (e.g. color Doppler ultrasound [CDUS]) and the incorporation of modern imaging modalities for diagnosis and monitoring of large-vessel vasculitis (computed tomography angiography [CTA] and positron emission tomography–computed tomography [PET-CT]).

Efficient and rapid diagnosis of GCA is crucial for preventing further morbidity and reducing the incidence of irreversible damage and complications, e.g. ischemic visual loss.^6^

In this article, we review state-of-the-art approaches for the diagnosis, monitoring, and treatment options of GCA.

## DIAGNOSIS

### Temporal Artery Biopsy

Given that GCA was previously considered limited to the inflammation of the cranial arteries, temporal artery biopsy (TAB) used to be considered the diagnostic “gold standard.”

Temporal artery biopsy is an invasive procedure with a possible complication rate of up to 0.5%, including facial nerve injury,^7^,^8^ ptosis,^9^ and, rarely, stroke.^10^ Even when optimally performed by a skilled surgeon, TAB lacks sensitivity compared to other diagnostic methods (39% versus 54% for CDUS),^11^ probably as a result of skip lesions,^12^ antecedent use of high-dose corticosteroids, or limitation of GCA to extracranial arteries with no cranial arteries involved.

Temporal artery biopsy should be obtained from the temporal artery on the more symptomatic side. The additional yield of contralateral artery biopsy ranges between 3% and 13%.^13^,^14^ The optimal length of the biopsied specimen is a matter of debate as some studies recommend an optimal length cut-off of 0.7–1.5 cm,^15^,^16^ while others concluded that there was no difference in the mean biopsy size between positive and negative samples.^17^,^18^

**Figure 1:**
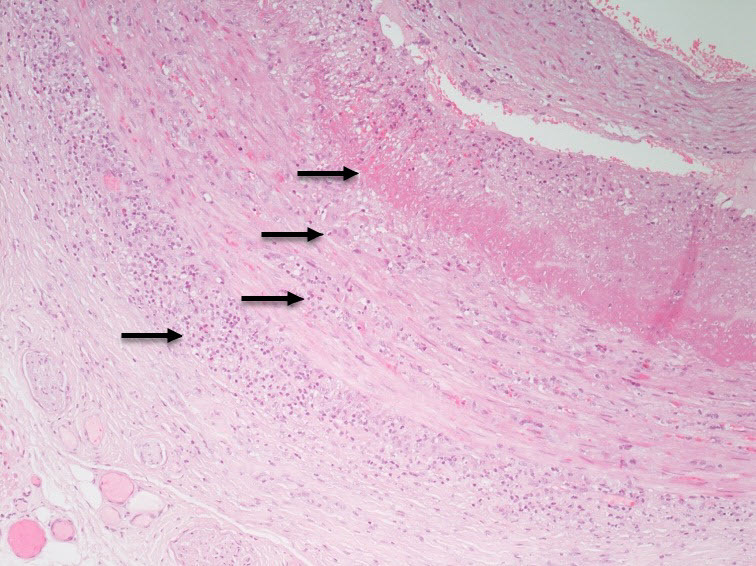
Transmural Inflammatory Infiltrate, Lymphohistiocytic Involving Intima, Elastic Internal Membrane, Media, and Adventitia (Arrows). Photo courtesy of Michael Lurie, M.D.

Several histological patterns of inflammatory changes can be suggestive of GCA, with panarteritis infiltrated by lymphocytes and macrophages being the most frequent (Figure 1), often with fragmented internal elastic lamina. Giant cells and histiocytes are found in 75% of specimens. Other less frequent inflammatory patterns in GCA include vasa vasorum vasculitis, in which inflammation is limited to the adventitial vasa vasorum without extension to the media.^19^,^20^

Interestingly, numerous studies reported giant cell presence in temporal artery biopsy as a strong predictor of ophthalmic complications and visual loss.^21^–^23^

Although the interpretation of histology findings in GCA seems straightforward, concerns were raised regarding poor inter-rater reliability among pathologists looking at the same TAB.^11^ Nevertheless, and despite its limitations, TAB is still the preferred diagnostic method in North America, probably due to lack of expertise in other diagnostic modalities, e.g. CDUS.^24^

### Color Doppler Ultrasound

Color Doppler ultrasound was introduced as a diagnostic modality for GCA in 1997 by Schmidt et al., who reported sonographic signs of inflammation in the temporal artery in 22 of 30 GCA patients.^25^ Being feasible and non-invasive, in the last two decades, CDUS has become the imaging modality of choice for diagnosing GCA in many centers worldwide. Moreover, dedicated, cost-effective fast-track GCA clinics utilizing CDUS for rapid diagnosis and, subsequently, treatment initiation of GCA have been proven to reduce the risk of visual complications significantly.^6^,^26^,^27^ In keeping with this, the trend towards visual loss has decreased considerably in the last decade, thanks to the early diagnosis of GCA and the early introduction of targeted treatments.^28^

Color Doppler ultrasound allows inspection of the complete course of each temporal artery and other arteries, including axillary, facial, occipital, and vertebral arteries, which contributes vastly to the accuracy and the yield of the diagnostics with an estimated sensitivity of 54%–94%.^11^,^29^

In patients suspected of GCA, CDUS of the temporal artery tree on both sides should be performed as soon as possible by an experienced examiner using a machine equipped with a high-frequency linear or hockey stick probe (15 MHz or more). Scanning axillary and subclavian arteries adds about 10% to the diagnostic yield of ultrasound because some patients with GCA have isolated extracranial vasculitis.^30^

**Figure 2:**
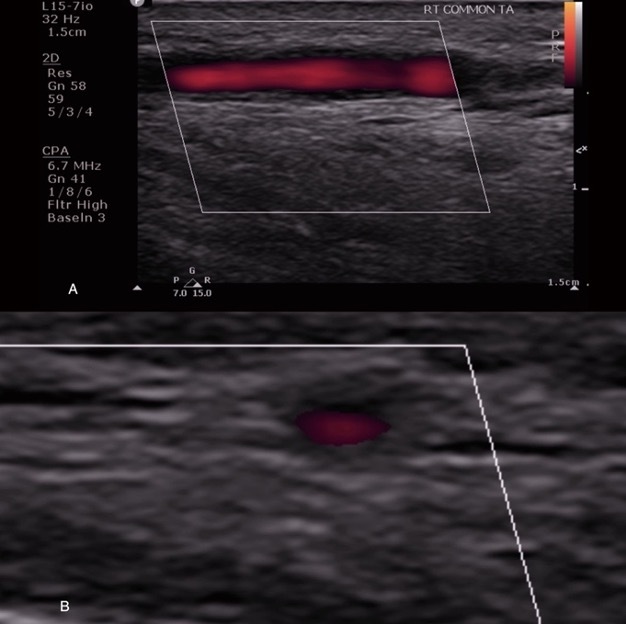
Longitudinal (A) and Transectional “Halo Sign” (B) of Frontal Branch of Right Temporal Artery in a 76-Year-Old Patient with GCA Showing Thickening of Arterial Wall.

In earlier reports, the cutoff of intima-media thickness of the artery wall representing active inflammation in the temporal and axillary artery was 0.35 mm and 1.0 mm, respectively (Figure 2A),^31^ but a recent large study proposed intima-media thickness cut-off values ≥0.4 mm for temporal, facial, and occipital arteries in order to further improve the diagnostic utility of CDUS.^32^ An additional and unique finding seen in CDUS, indicative of active inflammation, is the “halo” sign (Figure 2B), which is a hypoechoic halo around the lumen of the temporal artery.^33^

A low percentage (4%) of false positive halo signs has been reported in the literature in patients eventually diagnosed with amyloidosis, T cell lymphoma, atherosclerosis, and other forms of vasculitis as well.^34^–^36^

## ADVANCED IMAGING MODALITIES

The utility and importance of computerized tomography angiography (CTA), magnetic resonance angiography (MRA), and fluorodeoxyglucose positron emission tomography (FDG-PET) for the diagnosis of large-vessel vasculitis (LVV) were acknowledged in the new 2022 EULAR/ACR GCA classification criteria.^4^ A substantial percentage, from one-third and up to half of GCA patients, have extracranial LVV, depending on the imaging modality used.^37^–^39^

Large-vessel vasculitis involvement includes the aorta and major branches, e.g. axillary, subclavian, and, less frequently, mesenteric and iliac arteries.

**Figure 3:**
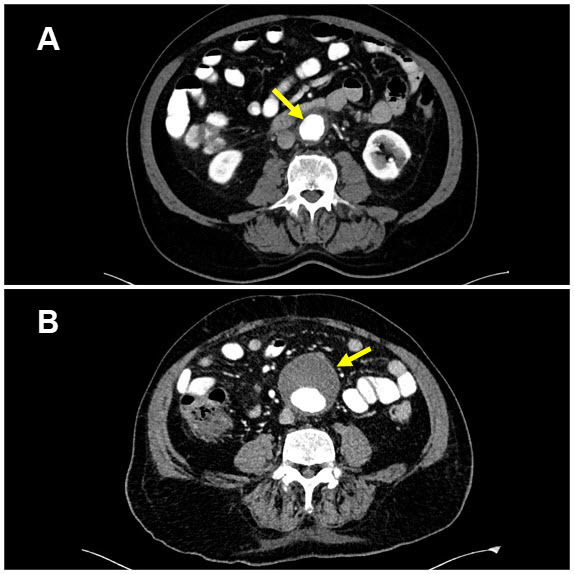
Color Doppler Ultrasound (CTA) Images of a Giant Cell Arteritis Patient. **(A)** A 2017 CTA showing active vasculitis of the abdominal aorta with aortic wall thickening (arrow); patient age 68 years. **(B)** The same patient in 2021 showing an aortic aneurysm (arrow); patient age 72 years.

Both CTA and MRA provide detailed information about the arterial lumen and wall. They can provide valuable input regarding the extent of involvement in medium and large vessels manifesting as concentric thickening of the arterial wall, dilatations, or potentially fatal aneurysms (Figure 3).

Magnetic resonance angiography is potentially more sensitive than CTA as it demonstrates vessel wall edema/contrast enhancement of inflamed arteries. Moreover, without an experienced vascular sonographer, high-resolution MRA can be utilized as an alternative for CDUS of temporal arteries since both modalities have comparable sensitivity and specificity (69%–73% and 88%–91%, respectively).^40^,^41^

Three-dimensional MRI black blood is a newer sequence with multiplanar and curved reconstructions designed to evaluate better the tortuous intracranial arteries^42^ and has a high spatial resolution for diagnosis of both cranial and extracranial vasculitis, harvesting promising results with reported sensitivity and specificity of 80% and 100%, respectively.^43^–^45^

**Figure 4:**
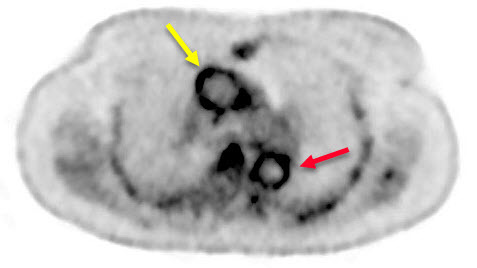
A PET-CT Image of GCA Patient with Active Vasculitis of Thoracic Aorta Showing Increased FDG Uptake in Arterial Wall of Both the Ascending (Yellow Arrow) and Descending (Red Arrow) Aorta.

Often combined with CT for optimal anatomic allocation, FDG-PET detects glucose reuptake in the walls of inflamed arteries (Figure 4). The imaging tool PET-CT is helpful for diagnosing and monitoring LVV^46^ and has been most recently proven valuable in detecting vasculitis in relatively small cranial arteries.^47^ When compared to CTA in 24 patients with GCA, PET-CT had comparable sensitivity but higher specificity and positive predictive value (66.7% versus 73.3%, 100% versus 84.6%, 100% versus 84.6%, respectively).^48^ The main pitfalls in PET-CT remain the lack of standardization criteria for assessing LVV,^49^ high radiation dose, and low availability. Moreover, specificity may be influenced by atherosclerotic lesions in elderly patients being misinterpreted as active vasculitis. However, this may be the population that mostly benefits from PET-CT as it is the modality of choice for ruling out malignancies that are not unusual in GCA patients.

The best imaging modality for assessment of LVV in GCA remains a matter of debate and depends on the clinical situation, local availability, and expertise.^50^

## DISEASE MONITORING

Once considered a disease resolving in 1–2 years in most patients,^51^ GCA has evolved to be a chronic and multisystem disease in which more than half of the patients experience flares.^52^,^53^ Hence, monitoring disease activity and response to treatment, along with identifying vascular complications, e.g. an aneurysm, is more vital than ever. Monitoring inflammatory markers and clinical state is not satisfactory since the introduction of biological therapies can potentially normalize the laboratory findings without achieving remission. Moreover, the activity of LVV can be indolent and subclinical.

Color Doppler ultrasound has been validated as a reliable modality and should be the tool of choice for monitoring cranial GCA considering its accessible, radiation-free, and non-invasive nature. Once treatment is initiated, the halo sign and intima-media thickness are reported to shrink within seven days, eventually disappearing after eight weeks in most patients,^54^–^56^ while recurrence of halo predicts flare with a high inter-rater agreement of 0.91.^57^ Moreover, the number of temporal artery segments with halo correlates with erythrocyte sedimentation rate, C reactive protein, and Birmingham Vasculitis Activity Score.^58^

The data concerning the role of MRA and CTA for monitoring GCA activity have been rather confusing since structural changes in vessel walls do not always indicate active disease but rather fibrotic and regenerative changes.^59^ Data from small studies showed that imaging characteristics in MRA often did not parallel that of laboratory or clinical parameters, although wall thickness significantly decreased at follow-up in 12 patients with LVV treated with biological therapies.^60^

Repetitive PET-CT scans are associated with a substantial dose of radiation, but when attempted in treated GCA patients it showed a decline in FDG uptake compared with pre-treatment uptake.^61^ Meanwhile, data from patients with clinically controlled GCA revealed long-term persistent vascular uptake on repeated PET-CT in >80% of our GCA patients with large-vessel inflammation and clinical-biological controlled disease.^62^ Similar results were reproduced in a prospective study of patients with LVV allegedly in clinical remission who underwent serial PET-CT scans that were interpreted as active vasculitis in 58% of patients and those who were more likely to relapse.^63^ These data support the hypothesis that persistent FDG uptake may reflect smoldering inflammatory activity, but we are yet to articulate specific criteria to guide treatment decisions.

Future novel biomarkers may aid in ameliorating GCA disease activity monitoring. Serum amyloid A1 and A2 and complement factor H were higher in patients with active disease and receiving prednisolone therapy. In addition, the haptoglobin blood test level seems to be higher and fibrinogen levels lower in patients with active disease taking tocilizumab.^64^,^65^

## TREATMENT

Vascular complications of GCA can be abrupt and irreversible, e.g. visual loss and cerebrovascular accidents, but these are not inevitable if GCA is diagnosed early and treated appropriately. Data from several studies have pointed out that when a visual loss occurs due to GCA, real visual improvement occurs only rarely, in an estimated 5% of patients.^66^,^67^

Patients with suspected GCA should be referred to fast-track GCA clinics to facilitate rapid diagnosis and, subsequently, full-dose treatment. In contrast, patients can discontinue steroids at once, avoiding needless treatment if GCA is excluded.

Although treatment of giant cell arteritis has evolved in recent years from glucocorticoids (GC) and broad-spectrum immunosuppressive agents to targeted therapies, the cornerstone of treatment in GCA remains GC.

When started at a high dose of 1 mg/kg, GC induces rapid improvement in both clinical symptoms and inflammatory markers, minimizing the odds of GCA complications. Hence, GC should be initiated once GCA is suspected and prior to a definite diagnosis. The main goal of high-dose GC is to induce remission and should be maintained for four weeks or until symptoms subside.^68^ Glucocorticoids should be slowly tapered after that, by 10 mg every two weeks until 20 mg/day is reached, then reduced by 2.5 mg every 2–4 weeks to 10 mg and afterward by 1 mg every 1–2 months according to clinical response. Despite controversies about whether intravenous GC are more effective than the oral route when ischemic ophthalmic involvement is suspected, patients are frequently treated with a high dose of 1000 mg i.v. methylprednisolone for three consecutive days to prevent further visual damage.^69^

About half of GCA patients experience at least one flare in the first year,^70^ even with optimal high-dose GC tapering protocol. Relapses are usually treated by escalating GC doses 10–15 mg above the previous effective dose, thus increasing the cumulative GC dose and the likelihood of GC long-term side effects, e.g. osteoporosis, diabetes mellitus, cataract, and cardiovascular events. The burden of GC in GCA patients is so substantial that an estimated 86% of patients suffer at least one GC-related side effect and 58% more than one when followed for a median of 10 years.^71^

The toxicity of long-term GC treatment in GCA led to studies evaluating several disease-modifying antirheumatic drugs (DMARDs) showing modest impact, if any. A meta-analysis of three small randomized, double-blind, placebo-controlled trials (RCTs) found that when treated with methotrexate, GCA patients experienced relative reduction in the risk of a first and second GCA relapse and were exposed to lower cumulative GC doses.^72^ Nevertheless, methotrexate should be preferred over other DMARDs as recommended by ACR^24^ and French^73^ guidelines for management of GCA and LVV. Retrospective case series showed the potential benefit of leflunomide in patients with refractory GCA, with partial or complete remission.^74^,^75^ Azathioprine demonstrated a modest effect on disease activity in GCA patients when evaluated in a retrospective study in 18/28 patients; 10 patients experienced azathioprine’s serious side effects, leading to treatment discontinuation in 7 cases.^76^

When evaluated, RCTs of tumor necrosis factor-alpha antagonists failed to show efficacy in GCA.^77^–^79^ An essential role is played by interleukin-6 (IL6) in the pathogenesis of GCA, and elevated circulating levels of IL6 have been reported in patients with active disease.^80^ Nevertheless, studies of small series of patients with successfully treated GCA were published only in 2011, in parallel to the introduction of targeted anti-IL6 therapies.^81^–^83^ In 2017, a 52-week GiACTA study demonstrated significant clinical responses and cumulative GC dose reduction following weekly and every-other-week administration of subcutaneous tocilizumab (TCZ) compared to placebo arms where GC were tapered within 26 and 52 weeks.^70^ The GiACTA extension study reports prolonged remission in about half of the patients previously receiving TCZ during the subsequent follow-up period compared to placebo.

Interestingly, despite clinical differences between real-life patients undergoing TCZ treatment and those included in the GiACTA trial (real-life patients were older with longer disease duration and higher values of erythrocyte sedimentation rate (ESR), TCZ was equally effective in both GiACTA trial and clinical practice patients.^84^

As a result of these studies, tocilizumab became the first biologic to receive US Food and Drug Administration approval for GCA. Subsequently, ACR recommends tocilizumab with GC as first-line treatment for GCA, while EULAR recommends it as second-line therapy.^24^,^68^

We recommend starting GCA patients initially with GC and reserving TCZ for relapsing disease or GC toxicity. Once on remission with TCZ and GC treatment, GC should be tapered within 26 weeks and discontinued. If remission is maintained, tocilizumab is often continued as monotherapy for 18–24 months. Given the fact that GCA relapse is still frequent following discontinuation of TCZ (about half of the patients),^85^ we recommend tapering TCZ to once every other week for an additional year before complete discontinuation.

An open-label, single-arm study performed with relapsing patients with GCA suggested the clinical benefit of ustekinumab.^86^ Granulocyte-macrophage colony-stimulating factor (GM-CSF) receptor antagonist mavrilimumab showed efficacy in 42 GCA patients compared to GC for time to flare and sustained remission after 26 weeks.^87^

Abatacept blocks the engagement of CD28 with its ligand thereby inhibiting T cell activation. When administered to newly diagnosed or relapsing GCA patients, relapse-free survival at 12 months was 48% for those receiving abatacept (anti CTLA-4) and GC and 31% for those receiving GC alone (*P*=0.049).^84^

New promising targeted therapies, e.g. Janus-activated kinase inhibitors and IL17/IL23 pathway inhibitors, are under investigation in ongoing trials.

## CONCLUDING REMARKS

Giant cell arteritis is a chronic disease in which relapses are common and long-term monitoring is required. The diagnosis and treatment of GCA has been revolutionized with the utilization of innovative imaging modalities and the introduction of IL6 blockage with TCZ. Despite the obvious advantages of PET-CT and MRA in GCA, these methods lack accuracy when used to distinguish active LVV from remission, especially when TCZ treatment significantly reduces acute phase reactants (erythrocyte sedimentation rate and C-reactive protein) regardless of clinical remission. Consequently, the clinical judgement of the treating rheumatologist will still have the major role in assessing disease activity in LVV.

## Abbreviations

CDUS: color Doppler ultrasound
CTA: computed tomography angiography
FDG-PET: fluorodeoxyglucose positron emission tomography
GC: glucocorticoids
GCA: giant cell arteritis
IL6: interleukin-6
LVV: large-vessel vasculitis
MRA: magnetic resonance angiography
PET-CT: positron emission tomography–computed tomography
TAB: temporal artery biopsy.
